# Risk factors and outcomes of conversion in minimally invasive distal pancreatectomy: a systematic review

**DOI:** 10.1007/s00423-020-02043-2

**Published:** 2020-12-10

**Authors:** A. Balduzzi, N. van der Heijde, A. Alseidi, S. Dokmak, M. L. Kendrick, P. M. Polanco, D. E. Sandford, S. V. Shrikhande, C. M. Vollmer, S. E. Wang, H. J. Zeh, M. Abu Hilal, H. J. Asbun, M. G. Besselink, on behalf of the International Evidence-based Guidelines of Minimally Invasive Pancreas Resection Group

**Affiliations:** 1 Department of Surgery, University Hospital, Verona, Italy; 2 Department of Surgery, Southampton University Hospital, Southampton, UK; 3 Department of Surgery, Cancer Center Amsterdam, Amsterdam UMC, University of Amsterdam, Amsterdam, The Netherlands; 4 Department of Surgery, University of California, San Francisco, CA USA; 5 Department of Surgery, Beaujon Hospital, Paris, France; 6 Department of Surgery, Mayo Clinic, Rochester, MN USA; 7 Department of Surgery, UT Southwestern Medical Center, Dallas, TX USA; 8 Department of Surgery, Washington University, St. Louis, MO USA; 9 Department of Surgery, Tata Memorial Hospital, Mumbai, India; 10 Department of Surgery, University of Pennsylvania, Philadelphia, PA USA; 11 Department of Surgery, Taipei Veterans General Hospital and National Yang-Ming University, Taipei, Taiwan, Republic of China; 12 Department of Surgery, University of Pittsburgh Medical Center, Pittsburgh, PA USA; 13 Department of General Surgery, Istituto Ospedaliero Fondazione Poliambulanza, Brescia, Italy; 14 Hepatobiliary and Pancreas, Miami Cancer Institute, Miami, FL USA

**Keywords:** Laparoscopic distal pancreatectomy, Robotic distal pancreatectomy, Conversion to open surgery, Conversion, Minimally invasive distal pancreatectomy

## Abstract

**Purpose:**

The reported conversion rates for minimally invasive distal pancreatectomy (MIDP) range widely from 2 to 38%. The identification of risk factors for conversion may help surgeons during preoperative planning and patient counseling. Moreover, the impact of conversion on outcomes of MIDP is unknown.

**Methods:**

A systematic review was conducted as part of the 2019 Miami International Evidence-Based Guidelines on Minimally Invasive Pancreas Resection (IG-MIPR). The PubMed, Cochrane, and Embase databases were searched for studies concerning conversion to open surgery in MIDP.

**Results:**

Of the 828 studies screened, eight met the eligibility criteria, resulting in a combined dataset including 2592 patients after MIDP. The overall conversion rate was 17.1% (range 13.0–32.7%) with heterogeneity between studies associated with the definition of conversion adopted. Only one study divided conversion into elective and emergency conversion. The main indications for conversion were vascular involvement (23.7%), concern for oncological radicality (21.9%), and bleeding (18.9%). The reported risk factors for conversion included a malignancy as an indication for surgery, the proximity of the tumor to vascular structures in preoperative imaging, higher BMI or visceral fat, and multi-organ resection or extended resection. Contrasting results were seen in terms of blood loss and length of stay in comparing converted MIDP and completed MIDP patients.

**Conclusion:**

The identified risk factors for conversion from this study can be used for patient selection and counseling. Surgeon experience should be considered when contemplating MIDP for a complex patient. Future studies should divide conversion into elective and emergency conversion.

## Introduction

Minimally invasive distal pancreatectomy (MIDP) includes both laparoscopic and robotic distal pancreatectomy. Several reports suggest that MIDP is associated with lower intraoperative blood loss, shorter time to start of oral intake and normal gastrointestinal function, shorter time to functional recovery, and a shorter hospital stay compared with open distal pancreatectomy (ODP) [1–10]. This has been confirmed by two randomized trials on MIDP vs. ODP—the LEOPARD and LAPOP trials [11, 12]. MIDP, however, remains a technically challenging operation, as shown by the high conversion rates, which range widely from 2 to 38%, even in high-volume centers [7, 13–15].

Several studies focused on postoperative morbidity and mortality, as well as oncological outcomes following MIDP. However, data regarding risk factors for conversion and outcomes after conversion are lacking. Conversion, especially when performed as an emergency, may negatively affect short-and long-term outcomes, as shown previously for liver surgery [16]. More data are needed to clarify risk factors and the impact of conversion on outcomes for MIDP. These findings may be relevant not only when comparing surgical series, as patient characteristics may differ between centers and countries, but also for patient selection and counseling for MIDP [17].

To the best of our knowledge, there are no existing systematic reviews that focused on risk factors for conversion in MIDP. The aim of this study was to systematically assess risk factors for conversion during MIDP and the impact of conversion on postoperative outcomes.

## Materials and methods

This systematic review was performed according to the Preferred Reporting Items for Systematic Reviews and Meta-Analysis (PRISMA) guidelines [18] as part of the 2019 Miami International Evidence-Based Guidelines on Minimally Invasive Pancreas Resection (IG-MIPR) [19] and was reported according to the Cochrane Handbook for Systematic Reviews of Interventions [20, 21].

### Literature search

A systematic literature search was conducted with the assistance of a clinical librarian according to the gold standard for systematic reviews in surgery [22]. The PubMed, Embase, Web of Science, and Cochrane databases were searched until 13 May 2020. Search terms were based on approach (e.g., minimally invasive surgery), procedure (distal pancreatectomy), and on the factors associated with conversion during minimally invasive distal pancreatectomy. The search on PubMed was as follows: “Pancreatectomy”[Mesh] OR “Pancreatic Diseases/surgery”[Mesh] OR pancreat*[tiab]) AND (“Minimally Invasive Surgical Procedures”[Mesh] OR “Laparoscopy”[Mesh] OR “Robotic Surgical Procedures”[Mesh] OR laparoscop*[tiab] OR robotic[tiab] OR robot-assisted[tiab] OR minimally invasive[tiab] OR minimal invas*[tiab] OR hybrid[tiab]) AND (“Conversion to Open Surgery”[Mesh] OR conversion[tiab].

### Eligibility criteria

Studies were included if they reported on factors associated with conversion in MIDP. Studies in languages other than English, duplicates, editorials, and studies on children were excluded. If several studies used the same dataset, only the most recent study was used.

### Study selection

Two authors (*AB* and *NH*) independently screened the identified studies. All references of included articles were manually screened for possible additional studies. The first selection was performed based on the title and abstract. Subsequently, the same authors independently performed an assessment of the full text. Any disagreement between the authors was resolved through discussion until a consensus was reached.

### Assessment of methodological quality

The included studies were critically appraised independently by two authors (*AB and NH*). A quality assessment of the selected studies was performed using the Scottish Intercollegiate Guidelines Network (SIGN) methodology [23]. SIGN was established for the development of evidence-based clinical guidelines. Each study type was assessed with a corresponding checklist, resulting in a quality level of high (++), acceptable (+), low (−), or unacceptable (reject).

The risk of bias was assessed according to the Newcastle Ottawa scale (NOS) for all studies, since no randomized controlled trials (RCT) were expected to be included. A maximum of nine points could be granted, divided equally over three categories—“selection of patients,” “comparability,” and “outcome of study participants.” Studies with a NOS score of ≤ 5 were considered exhibiting a high risk for bias.

### Data extraction

Data extraction was performed according to a predefined evidence table, which was then cross-checked independently by two of the authors (*AB* and *NH*). Extracted variables included study design; study period; sample size; patient characteristics (age, sex, BMI, tumor size, diagnosis);operative outcomes; conversion; intraoperative blood loss; operative time; R0 resection margin; and postoperative outcomes such as Clavien–Dindo grade ≥ 3 complications, clinically relevant postoperative pancreatic fistula according to the International Study Group on Pancreatic Surgery (ISGPS) definition [24], and length of hospital stay.

## Results

### Search results

**Fig. 1:**
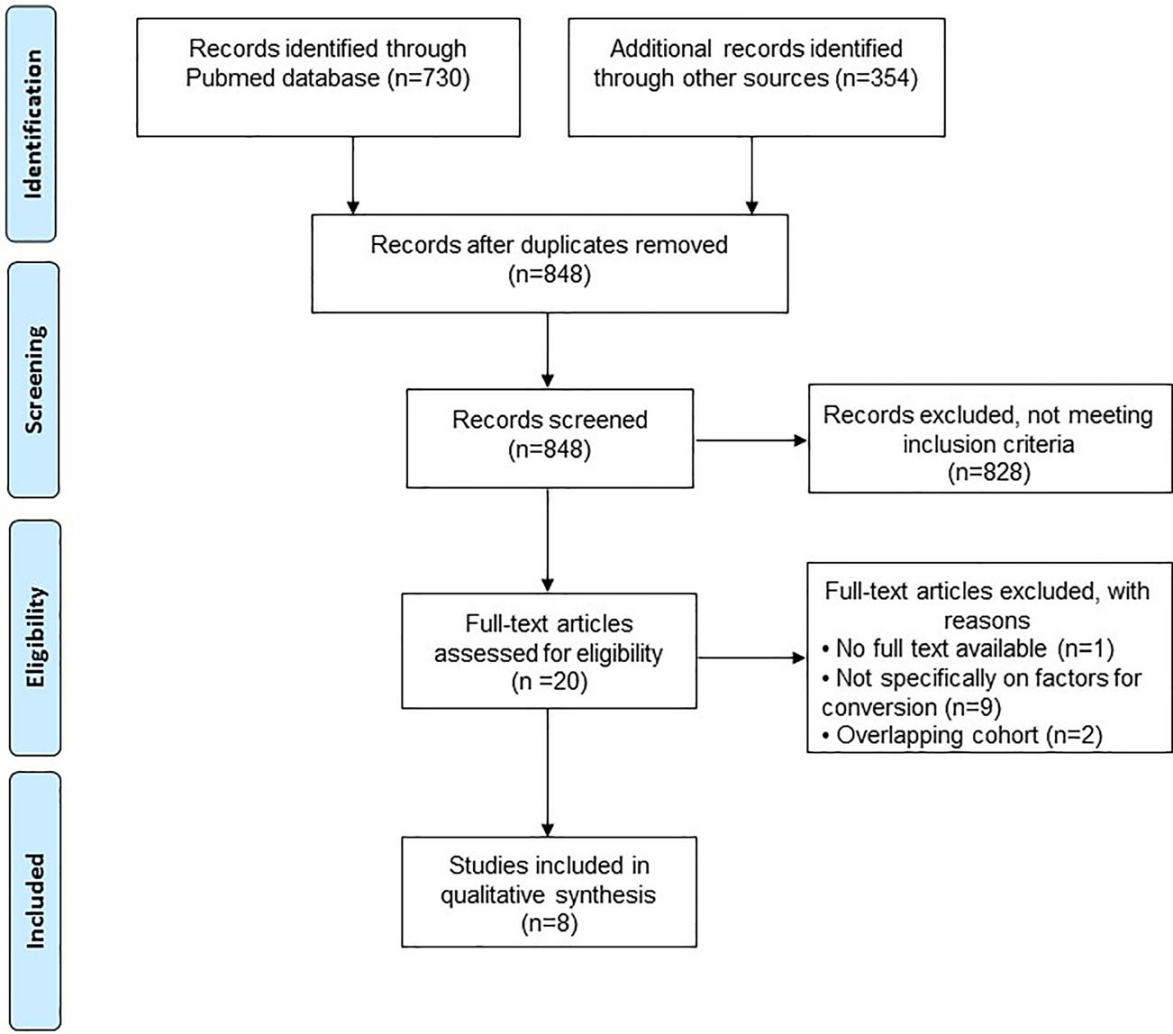
PRISMA flowchart of all included studies

A total of 848 studies were identified after duplicates were removed. After the screening of titles and abstracts, 20 studies remained for full-text assessment, of which seven met the eligibility criteria outlined for this review. One additional study was identified after screening the references of the included studies [25]. The PRISMA study selection flow diagram is shown in Fig. 1.

### Methodological quality

Using the SIGN methodology, two (25%) of eight studies were considered low quality [26, 27] and the remainder were considered acceptable quality. One (12.5%) study had a high risk of bias (≤ 5 NOS score) because detailed information on patient follow-up was lacking [26]. Only one study used a form of matching [28].

### Definition of conversion

The characteristics of the included studies are shown in Table 1. Half of the studies did not include a clear definition of conversion [27, 29–31]. Three studies stated that an open conversion was defined as a resection performed via a laparoscopic, robotic, or hand-assisted approach but that an open incision was needed to complete the resection regardless of incision size [25, 26, 32]; two of these studies specifically stated that conversion to a hand-assisted approach was not considered a conversion [25, 26]. One study categorized conversions as either elective or emergency conversions, the latter due to unexpected events (e.g., bleeding), the former a result of unexpected findings such as tumor extensions to adjacent organs or vascular structures, difficulty in tumor exposure, and adhesions [28].

**Table 1:** Baseline characteristics of the included studies

Study	Study period	Patients (*n*)		Conversion rate (%)	Approach	Single or multicenter	Study design	Case matching
		Converted	Total					
Casadei et al.	2004–2016	13	68	19.1	Laparoscopic	Single center	Retrospective	No
Goh et al.	2006–2015	10	40	25.0	Laparoscopic	Single center	Retrospective	No
Hanna et al.	2006–2012	9	57	15.8	Robotic	Single center	Retrospective	No
Hua et al.	2007–2015	31	211	14.7	Both	Single center	Prospective	No
Lee et al.	2000–2013	55	168	32.7	Both	Single center	Prospective	No
Lof et al.	2011–2015	68	345	19.7	Both	Multicenter	Retrospective	Yes
Nassour et al.	2014–2015	231	1512	15.3	Both	Multicenter	Prospective	No
Partelli et al.	2015–2018	25	191	13.0	Laparoscopic	Two centers	Retrospective	No
Total	2000–2018	442	2592	17.1				

### Indication for conversion

Five (62.5%) studies elaborated on the indications for conversion [25–28, 30]; another study included different types of pancreatic resections but did not specify the indications for distal pancreatectomy separately [27]. All indications for conversion in MIDP from the included studies are listed in Table 2. When combining the indications for conversion of all included studies, vascular involvement was the cause that most often led to a conversion (*n* = 40, 23.7%), followed by concern for oncological margin (*n* = 37, 21.9%) and bleeding (*n* = 32, 18.9%).

**Table 2:** Indications for conversion in minimally invasive distal pancreatectomy

	Laparoscopy *n* (%)	Robot *n* (%)	Total *n* (%)
*Goh* et al.	10	–	10
Oncological concerns	4 (40.0)	–	4 (40.0)
Adhesions	3 (30.0)	–	3 (30.0)
Bleeding	3 (40.0)	–	3 (40.0)
*Hua* et al.	36	–	36
Obesity	10 (27.8)	–	10 (27.8)
Adhesions	10 (27.8)	–	10 (27.8)
Oncological concerns	8 (14.5)	–	8 (14.5)
Vascular involvement tumor	6 (16.7)	–	6 (16.7)
Bleeding	2 (5.6)	–	2 (5.6)
*Lee* et al.	41	14	55
Obesity	13 (31.7)	4 (28.6)	17 (30.9)
Vascular involvement tumor	12 (29.3)	2 (14.3)	14 (25.5)
Adhesions	4 (9.6)	2 (14.3)	6 (10.9)
Bleeding	5 (12.2)	–	5 (9.1)
Oncological concerns	3 (7.3)	2 (14.3)	5 (9.1)
Technical inability to proceed minimally invasive	3 (7.3)	2 (14.3)	5 (9.1)
Pancreatic inflammation	–	2 (14.3)	2 (5.5)
Varices	1 (2.4)	–	1 (1.8)
*Lof* et al.	67	1	68
Bleeding	22 (32.8)	–	22 (32.4)
Vascular involvement tumor	20 (29.9)	–	20 (29.4)
Oncological concerns	19 (28.4)	1 (100)	20 (29.4)
Adhesions	4 (6.0)	–	4 (5.9)
Poor visualization tumor	2 (3.0)	–	2 (2.9)
*Total*	154	15	169
Vascular involvement tumor	38 (24.7)	2 (13.3)	40 (23.7)
Oncological concerns	34 (22.1)	3 (20.0)	37 (21.9)
Bleeding	32 (20.8)	–	32 (18.9)
Obesity or poor visualization tumor	25 (16.2)	4 (26.7)	29 (17.2)
Adhesions	21 (13.6)	2 (13.3)	23 (13.6)
Other	4 (2.6)	4 (26.7)	8 (4.7)

### Risk factors for conversion

Six studies assessed the risk factors for conversion [25, 28–32]. An overview of the independent risk factors for conversion from multivariate analyses can be found in Table 3. Three studies assessed both laparoscopic and robotic approaches, two of which found a significantly lower conversion rate for the robotic approach [28, 29]. Recurrent preoperative risk factors for conversion were obesity, a high BMI (BMI > 30) [28, 29], or a high amount of visceral fat [25], as well as a preoperative suspicion of a malignancy [30–32]. Intraoperative risk factors included multi-organ resection or a resection extending to neighboring organs [28, 30, 32] and tumor proximity to vascular structures [28, 31]. Other risk factors found were a low preoperative albumin level, a current smoking habit, and chronic pancreatitis [29]. In a univariate logistic regression analysis, Partelli et al. reported that age and the pancreatic resection line (portal vein vs. distal pancreas) are risk factors for conversion [31].

**Table 3:** Independent risk factors for conversion in minimally invasive distal pancreatectomy

Study	Preoperative risk factors	Intraoperative risk factors
Casadei et al.	None of the factors were significant in multivariable analysis	Extension of pancreatic resection
Goh et al.	Not analyzed	Not analyzed
Hanna et al.	Not analyzed	Not analyzed
Hua et al.	Preoperative diagnosis of malignant disease Surgeon LDP experience (≤ 15 cases)	Resection of other organs required
Lee et al.	None of the factors were significant in multivariate analysis	Visceral fat
Lof et al.	Tumor proximity to vascular structures (< 1 cm) in preoperative imaging	Not analyzed
Nassour et al.	Higher BMI Higher preoperative albumin level Current smoking habit Malignant T3/T4 disease Chronic pancreatitis	Laparoscopic approach
Partelli et al.	Tumor close to vessel (< 2 cm) on preoperative imaging	Not analyzed

### Surgeon procedure volume

Casadei et al. reported a cutoff to complete the learning curve for laparoscopic distal pancreatectomy (LDP) of 17 procedures. However, the learning curve cutoff was not correlated with the risk of conversion [32]. Two other studies analyzed surgeon experience in relation to conversion. Goh et al. defined a high-volume surgeon as one who performed > 5 LDPs and found a 10.5% conversion rate for high-volume surgeons vs. 38.1% for low-volume surgeons (*p* = 0.044) [26]. Hua et al. defined the case experience of surgeons as either low (< 15 LDP cases performed) or high (≥ 15 LDP cases performed). They reported a 10.3% conversion rate for highly experience surgeons vs. 20.2% for low experience surgeons (*p* = 0.042). In a multivariate analysis, surgeon experience was a significant independent risk factor for conversion (OR 0.32, 95% CI 0.12–0.85, *p* = 0.023) [30]. Partelli et al. defined a surgeon’s experience as high when they had performed at least 30 LDPs as first operator; 76% (*n* = 145) of all the cases in their study were with a highly experienced surgeon. Surgeon experience was not a significant risk factor for conversion in univariate regression in their study (OR 0.76, 95% CI 0.27–2.16, *p* = 0.609) [31].

### Outcomes after conversion

Nassour et al. [34] compared patient outcomes between those who had MIDP with those who underwent conversion in MIDP and found that the latter group had a longer mean length of hospital stay (mean 8 vs. 6 days, *p* < 0.001), higher re-operation rate (*n* = 15, 6.5% vs. *n* = 31, 2.4%), and higher 30-day mortality rate (*n* = 5, 2.2% vs. *n* = 4, 0.3%, *p* = 0.006). Converted MIDP also showed a higher rate of re-operation compared with ODP patients (*n* = 15, 6.5% vs. *n* = 49, 3.5%, *p* = 0.027).

Two other studies also compared the outcome for patients with a conversion with those who had a complete MIDP. The first found that patients with conversion had a higher rate of intraoperative blood loss and transfusion, with comparable pancreatic fistula rates and a longer hospital stay [27]. In contrast, the second found no differences in terms of operation time, blood loss, transfusions, pancreatic fistula, and length of hospital stay [26].

One study divided conversion into two categories—elective and emergency conversions [28]. A comparison was made between elective and emergency conversions and ODP patients with the use of propensity score matching. Compared to ODP patients, emergency converted patients exhibited a significantly longer operation time (median 285 vs. 240 mins, *p* = 0.013), higher intraoperative blood loss (median 850 vs. 400 mL, *p* = 0.002), greater need for blood transfusions (*n* = 9, 45.0% vs. *n* = 3, 6.0%, *p* < 0.001), and a higher rate of minor (*n* = 16, 13.1% vs. *n* = 10, 47.6%, *p* < 0.001) and overall morbidity (*n* = 29, 47.5% vs. *n* = 38, 31.1%, *p* = 0.030). In contrast, besides differences in average operative time and minor morbidity, there were no significant differences in postoperative outcome between elective converted MIDP and ODP patients [28].

## Discussion

MIDP is increasingly considered the standard approach for patients undergoing distal pancreatectomy in high-volume centers. In this systematic review focusing on risk factors for conversion in MIDP, we found a 17% overall conversion rate that was affected by several risk factors: smoking, high BMI, preoperative albumin level, malignant disease (T3/T4), chronic pancreatitis, surgeon experience with concurrent vascular resection, and multi-organ resection/extended pancreatic resection. These factors can be considered separately in the preoperative setting.

Numerous studies analyzed the risk factors for conversion in other types of minimally invasive gastrointestinal surgery such as cholecystectomy, nephrectomy, liver, and colorectal surgery [16, 33–39]. The main risk factors identified in these studies were high BMI [33, 38], past abdominal infections [37], past abdominal surgery [35], adhesions [34, 38, 39], diagnosis of malignant disease [40], and blood vessel anatomy [38].

Converted MIDP showed a longer operative time and higher intraoperative blood loss, re-operation rate, 30-day mortality, and overall complication compared with ODP. However, differentiating between elective and emergency conversion revealed that elective conversions seem to be comparable to ODP with regard to short-term outcomes, whereas emergency conversions are associated with worse outcomes.

Information regarding the timing of conversion and indication leading to it was often lacking. One might expect that an elective conversion is associated with a smaller, or absent, risk of increased operative time, blood loss, and additional morbidity. Only one study specifically assessed the difference in outcome between elective and emergency conversions and confirmed this hypothesis. Future studies should distinguish between elective and emergency conversions rather than judging conversion as a complication. Currently, a surgeon may lean toward persevering with the MIS approach because of the current bias toward considering conversion a failure. Such a surgical culture may play a role in delaying a conversion when it is needed and may turn an elective conversion into an emergency conversion, with the end result being higher morbidity.

Data concerning the minimum MIDP experience and annual volume per surgeon versus the risk of conversion in MIDP were scarce. The definitions of a “high-volume surgeon” varied widely between 5, 15, and 30 LDPs performed. Considering this range of definitions, it is difficult to compare outcomes between studies. The influence of surgeon experience on conversion was shown in a nationwide study on the impact of a training program that included a detailed description of the technique, video-training and on-site proctoring on MIDP. After the training program, the conversion rate for MIDP decreased from 38 to 8% (*p* < 0.001) [14]. According to the Miami guidelines on minimally invasive pancreatic resections, depending on the outcome used to assess the learning curve, 10–40 LDP cases are needed to reach proficiency [19]; however, an exact requirement is yet to be defined.

When assessing higher BMI as a risk factor for conversion, the included studies provided conflicting results. This discrepancy could be explained by the fact that BMI might not be an accurate measurement of obesity. It is known that the relationship between visceral fat and BMI differs between men and women; therefore, an intra-abdominal fat may be a better method for measuring obesity [41]. Moreover, not all included studies used a clear cutoff for high BMI or it was only specified as “higher BMI” or “BMI per unit increase.”

Two previous studies assessed the impact of obesity in MIDP. The first compared obese patients (BMI ≥ 30, *n* = 56) with normal weight (BMI < 25, *n* = 191) and overweight patients (BMI 25–29.9, *n* = 155) and concluded that conversion rates did not differ significantly across the three groups (BMI ≥ 30, *n* = 1; 1.8% vs. BMI < 25, *n* = 1; 0.5% vs. BMI 25–29.9, *n* = 5; 3.2%, *p* = 0.15) [42]. These outcomes were in line with another study that included 57 nonobese (BMI < 30) and 28 obese patients (BMI ≥ 30) who were undergoing robotic distal pancreatectomy. There was no significant difference in conversion rate between the two groups (BMI < 30: 5.3% vs. BMI ≥ 30: 3.5%; *p* = 0.071) [43]. However, both of these studies were retrospective, so there is a high risk of patient allocation bias and other possible confounding factors were not taken into account during the analysis (e.g., no multivariate logistic regression with conversion as a dependent variable was performed). Thus, with the existing literature, it remains unclear whether or not high BMI is a risk factor for conversion in MIDP.

The results of this study should be assessed with several limitations in mind. First, the number of included studies was low. Since variation may exist between centers and countries, for instance, regarding patients’ BMI and surgical volume, more multicenter, and preferably international, studies are needed. Additionally, a uniform cutoff for high-volume MIDP surgeons should be used in future studies to facilitate an assessment of the impact of surgeon experience and center volume on the risk of conversion. Second, the number of indications for MIDP has increased over time, probably due to the growing surgical experience. Third, due to the limited data available, we combined data from laparoscopic and robotic procedures, so it is unclear whether there are different risk factors for these two approaches. Fourth, we did not include Web of Science as a database in our search. Finally, definitions for conversion vary, making it difficult to compare study outcomes. Conversion should be categorized as either emergency or elective conversion to enable a robust comparison between the open approach and total MIDP.

In summary, this study aimed to provide an overview of the current literature for conversion in minimally invasive distal pancreatectomy. Although some risk factors were identified from the included studies, reaching definitive conclusions will require standardization of definitions and data collection protocols in future studies. Larger trials, adjusting for baseline characteristics by using either a form of matching or regression analysis to minimize selection bias, are needed to confirm the findings of this systematic review. Future studies should focus more on the indications for conversion by classifying converted patients into either the elective or emergency conversion group. The Miami guidelines on minimally invasive pancreatic resection recommend the creation of standardized databases to facilitate rigorous study and a deeper understanding of the reasons for conversion in MIDP and their effect on outcomes [19].
